# Insecticidal efficacy of nanomaterials used to control mosquito, Culex quinquefasciatus Say, 1823 with special reference to their hepatotoxicity in rats

**DOI:** 10.1042/BSR20220630

**Published:** 2022-07-27

**Authors:** Muhammad S.M. Shamseldean, Marwa M. Attia, Reda M.S. Korany, Nehal A. Othamn, Sally F.M. Allam

**Affiliations:** 1 Department of Zoology and Agricultural Nematology, Faculty of Agriculture, Cairo University, Giza 12613, Egypt; 2 Department of Parasitology, Faculty of Veterinary Medicine, Cairo University, Giza 12211, Egypt; 3 Department of Pathology; Faculty of Veterinary Medicine, Cairo University, Giza 12211, Egypt

**Keywords:** 1823, Chitosan nanoparticles, chitosan-silver nanocomposites, Culex quinquefasciatus Say, histopathology, immunohistochemistry, Lavandula officinalis nanoemulsion

## Abstract

The present study aimed to develop a novel methodology for controlling the mosquito larvae using different nanoparticles, with special reference to their effect on rats (a non-target mammalian model). The mosquito species of *Culex quinquefasciatus* was reared in the laboratory. Chitosan, silver nanoparticles and their combination as well as lavender (*Lavandula officinalis*) nanoemulsion with different concentrations were tested as biological insecticides against the mosquito larvae. Mammalian toxicity of the used nanoparticles were evaluated using 27 adult male rats, experimental rats were divided into 9 equal groups (*n*=3). The nanoparticles were added to the drinking water for 30 days. At the end of the study, blood and tissue samples were collected to assess the levels of the serum alanine aminotransferase and aspartate aminotransferase, different genes expression as interleukin 6 (IL-6) and IL-1β activity. Histopathological and immunohistochemical studies using two markers (TNF-α and BAX expression) were also applied. The LC_50_ and LC_90_ were recorded for each tested nanoparticles, and also the changes of the treated mosquito larvae cuticle were assessed using the scanning electron microscopy. Green nanoemulsion (*Lavandula officinalis*) was more effective than metal (silver) or even biodegradable (chitosan) nanoparticles in controlling of *Culex quiquefasciatus* mosquito larvae, and also it proved its safety by evaluation of the mammalian hepatotoxicity of the tested nanoparticles.

## Introduction

Mosquitoes are a large group of insects which belong to family Culicidae (order: Diptera). They transmit numerous diseases to both humans and animals [1]. Mosquitoes are the most dangerous insect pests responsible for millions of annual fatalities in the world [1,2]. They are playing an active role in transmitting deadly diseases such as Malaria of man and birds as well as life-threatening viruses such as ‘Chikungunya, Dengue fever, Yellow fever, Rift valley fever and Zika viruses’. All these diseases and more have a certain mosquito species as a vector of the disease causative agent and when female mosquitoes feed on either animal and/or human blood they transmit all these diseases. Most mosquito problems especially in developing countries such as Egypt are faced by applying synthetic chemical insecticides, and the excessive use of these polluted compounds has created markets for biopesticide products worldwide [3].

Different types of pesticides as chemical, natural and/or biological were used in controlling different stages of mosquitoes, but research work confirmed that some of these pesticides are pollutant to the environment, human, plant and/or animals causing several diseases. The mosquitoes have also developed different levels of resistance toward most of these control agents and they almost lost their efficacy as insecticides [4].

Insecticides resistance is widespread and urgent global problem. The number of insects with insecticide resistance has been rapidly increasing [5]. Resistance makes insecticides ineffective and leads to increase in their usage, which harms the non-target species and agricultural workers [6].

Now, there is an urgent need to develop novel and environmentally safe materials used to control mosquitoes. These materials should be biodegradable and target-specific insecticides [7]. Bioactive organic compounds produced by plants can act as repellents, food deterrents and/or growth inhibitors [8].

Nanoparticles can be used as effective pesticides and may also be incorporated into new formulations of insecticides and insect repellents without any hazard posed by the traditional chemical methods [9]. Most of them relied on nanomaterials prepared through the so-called green synthesis method, where extracts from plants, fungi, bacteria and even dead insects have been successfully employed to reduce and stabilize the nanoparticles [10].

Thus, the present work aimed to evaluate different nanoparticles (silver, chitosan and lavender) as an effective method for controlling the mosquitoe *Culex Quinquefasciatus* Say, 1823, also to assess their safety by evaluating their hepatotoxicity in rats as a non-target mammalian model.

## Materials and methods

### Establishing mosquito colony

Mosquito eggs of the mosquito species, *Culex quinquefasciatus* Say, 1823, were obtained from the Medical Entomology Research Institute (Dokki, Giza, Egypt). The identification of the mosquito to the species level was verified through MosKeyTool at the following website: https://www.medilabsecure.com/moskeytool.html [11]; an interactive identification key for mosquitoes of Euro-Mediterranean. The immature and mature stages of this mosquito species were reared according to the WHO, Attia et al. [2,12,13]. Immature stages of the mosquito were reared in enamel trays (each, 10 cm × 20 cm) that filled with de-chlorinated tap water. The larvae were fed Powdered biscuit + powdered milk+ Brewer’s yeast in a ratio of 2:1:1 at a temperature of 26 ± 2°C and a 12:12 h light and dark (L:D) photoperiod. Pupae were collected, transferred to plastic containers and kept inside mosquito cages (40 × 40 × 40 cm^3^) for adult emergence. After emergence, a 10% sugar solution in a cotton pad was provided for adults continuously. Mosquito females were blood fed on restrained pigeons placed inside the cages for egg production. Different immature mosquito stages were exposed separately to test the efficacy of the chosen nanoparticles [14].

### Tested nanoparticles and/or nanoemulsions

Chitosan, silver nanoparticles and its combination as well as lavender, *Lavandula officinalis* nanoemulsion were purchased from Nawah Scientific Research Company (Cairo, Egypt). All the nanoparticles were purchased characterized and certified with the following nano sizes according to the certificate of Nawah Scientific Research Company: (1) Chitosan nanoparticles were nearly spherical with an average diameter of 8–14 nm. The silver nanoparticles were spherical with an average diameter of 6.05–20.08 nm. Chitosan-silver nanocomposites varied from 40 to 60 nm. The silver nanoparticles were embedded inside and around the chitosan macromolecules. Meanwhile, the *Lavandula officinalis* nanoemulsion was 78–90 nm in diameter.

### Mortality bioassay

Different nanoparticles concentrations of chitosan, silver and its composites with chitosan as well as lavender nanoemulsion were tested. Each concentration was freshly prepared and applied in five replicates in distilled water. Ten highly active mosquito larvae (L_4_) were used to test each concentration. Insect larvae were immersed in 100 ml of each dilution for 20 min with continuous stirring of each diluent [2–15].

### Assessment of the insecticidal efficacy of the tested nanoparticles

All tested nanoparticles were removed at the end of each exposure period, and the stages were washed several times with de-chlorinated tap water, transferred to clean beakers and kept under observation for 24 h post-exposure for evaluation of mortality (%). The average mortality of the five replicates was used to calculate the efficacy. The experiments were carried out at the same time on the control larvae [14].

### Scanning electron microscopy examination of the tested mosquito larvae

Scanning electron microscope (SEM) was used to investigate the effect of different nanoparticles on the cuticle of the fourth instar mosquito larvae. After larval exposure to different concentrations of nanoparticles, all larvae were washed multiple times with buffered phosphate saline (pH 7.2) and labeled for their concentrations. The larvae were fixed for 24 h in chilled 2.5% glutaraldehyde, dehydrated in a serial upgraded ethanol degrees, dried in a CO_2_ critical point drier (Autosamdri-815, Germany), and mounted on stubs with a double sticky tape before being sputter coated with 20 nm gold (Spi-Module sputter Coater, U.K.) as detailed by Attia et al. [16] The larvae were imaged using a Scanning Electron Microscope (JSM 5200, Electron Probe Microanalyzer, JEOL, Japan) at the Applied Center for Entomonematodes (ACE), Agricultural Experimental Station, Faculty of Agriculture (Cairo University, Egypt).

### Monitored criteria

Pupation rate was estimated using the following equation: Pupation (%) = *A*/*B* × 100 (where, *A* = number of pupae, *B* = number of the tested larvae). Pupal mortality was indicated by a failure to respond to mechanical stimulation or failure to metamorphose into the adult stage. Pupal mortality percent was estimated using the following equation: Pupal mortality (%) = [(*A* − *B)/A*] × 100 (where, *A* = number of produced pupae, *B* = number of emerged adults). Adult emergence of men and women was counted and calculated using the following equation: Adult emergence (%) = *A*/*B* × 100 (where, *A* = number of emerged adults and *B* = number of tested pupae). These previous equations were calculated according to Finney [17].

### Evaluation of the toxicity of tested nanoparticles on rats as a mammalian model

#### Animal grouping and experimental design

Twenty seven adult male Sprague-Dawley rats (120–180 gm bwt) were obtained from the Faculty of Veterinary Medicine (Cairo University, Egypt). Animal experiment took place at Faculty of Veterinary Medicine, Cairo University, Egypt. Rats had ad libitum access to basal ration and tap water. All rats were acclimatized for 2 weeks before the beginning of the experiment. Animal handling and treatment procedures were conducted according to the Guidelines for the Care and Use of Laboratory Animals of the Faculty of Veterinary Medicine, Cairo University, Egypt and approved by the research ethics committee of the Faculty of Veterinary Medicine, Cairo University with approval number (VetCU12/10/2021/370). Rats were randomly distributed into nine equal groups (*n*=3). Group I (control) received distilled water, group II received 500 ppm chitosan nanoparticles, group III received 1000 ppm chitosan nanoparticles, group IV received 500 ppm silver nanoparticles, group V received 1000 ppm silver nanoparticles; group VI received 500 ppm chitosan–silver nanocomposite, group VII received 1000 ppm of chitosan–silver nanocomposite, group VIII received 500 ppm of lavender nanoemulsion, and group IX received 1000 ppm of lavender nanoemulsion. All tested nanoparticles were received orally in drinking water for 30 days. Concentrations were selected according to Sadek et al. [18] At the end of the experiment all animals were killed by cervical dislocation.

#### Blood and tissue sampling

At the end of the experimental period, blood samples were obtained from the retro-orbital venous plexus of the control and treated rats, blood was allowed to clot at room temperature before being centrifuged at 3000 rpm for 10 min to separate the serum, serum was stored at −20°C for the biochemical analysis [19]. Liver of the examined rats were aseptically dissected and stored at −80°C for gene expression. For histopathological and immunohistochemical studies, liver tissue was sampled and fixed in 10% buffered neutral formalin.

#### Biochemical evaluation

According to Reitman and Frankel [20], the effect of the tested nanoparticles on rat liver was assessed by monitoring the serum alanine aminotransferase ‘ALT’ and aspartate aminotransferase ‘AST’ levels. Kits for the biochemical analysis determinations were purchased from the Biodiagnostic Company (Dokki, Giza, Egypt).

#### Evaluation of interleukin-6 and IL-1β activity

Liver of the examined rats were aseptically dissected. Samples from three control rats were collected in the same manner and used as negative controls.

#### RNA isolation

The total RNA kits were used to isolate mRNA from 100 mg of liver tissue (Ambion, Applied Biosystems, Bedford, MA, U.S.A.). The sampled tissues were homogenized in Lysing Matrix D tubes using a FastPrep-24 homogenizer (MP Biomedicals, 2 cycles of 30 s at 6 m/s) (MP Biomedicals, Cairo, Egypt). Nanodrop was used to determine the purity and quantity of mRNA (Thermo Scientific, Waltham, MA 02451, U.S.A.). Following the manufacturer’s directions, 500 nanograms of mRNA were produced using DNaseI amplification grade (Invitrogen, Carlsbad CA, 92008, U.S.A.). The High-Capacity cDNA Archive Kit (Applied Biosystems, Bedford, MA, U.S.A.) was used to reverse transcribing the treated mRNA [21,22].

#### Quantitative real-time PCR protocol (*q*RT-PCR)

PCR primer sets for interleukin-6 and IL-1 specific for rats were constructed using sequences from the GenBank database (Table 1). GAPDH was utilized as a reference gene and to normalize the samples. The expression of the genes studied in this work was assessed on a different pool of cDNA derived from three non-infected rats who had previously been screened for parasites according to the methods described by Attia et al. [17] The PCR’s condition was followed according to Younis et al. [22] (Table 2).

**Table 1:** The sequences of the forward and reverse primer used in the quantitative real-time PCR

Gene	Sequence	Accession number	Reference
IL-6	F-5′-AGTAGTGAGGAACAAGCCAGAGC-3′	NM_012589	Qing He et al*.*, 2021
	R-5′-TTGGGTCAGGGGTGGTTATTG-3′		
IL-1β	F- 5′-GTGGCAATGAGGATGACTT-3′	XM_032902343	Qing He et al*.*, 2021
	R-5′-TGGGCTTATCATCTTTCAA-3′		
GAPDH	F-5-ACTTTGGTATCGTGGAAGGACTCAT-3	NM_001009784	Puech et al*.*, 2015
	R-5-GTTTTTCTAGACGGCAGGTCAGG-3		

**Table 2:** PCR cycling conditions

Steps	Temperatue	Time
Initial denaturation	95°C	10 min
40 cycles		
Denaturation	95°C	30 s
Annealing	60°C	30 s
Extension	72°C	45 s
Final extension	72°C	10 min

#### Histopathological examination

At the end of the experimental period, liver tissue specimens were collected from all experimental groups, fixed in neutral buffered formalin 10%, washed, dehydrated, cleared and embedded in paraffin. The paraffin embedded blocks were sectioned at 5-micron thickness and stained with hematoxylin and eosin [23] for histopathological examination. Stained sections were examined by a light microscope (Olympus BX50, Japan).

#### Histopathological lesion scoring

Histopathological alterations in liver were recorded and scored as, no changes (0), mild (1), moderate (2) and severe (3) changes, the grading was determined by percentage as follows: <30% changes (mild change), <30–50% (moderate change) and >50% (severe change) [24,25].

#### Immunohistochemistry of the liver tissue

Immunohistochemical analysis was carried out following the methods described by El-Maksoud et al [26] Tissue sections from liver were deparaffinized in xylene and rehydrated in graded alcohol. Hydrogen peroxide block (Thermo scientific, U.S.A) was added to block the endogenous peroxidase activity. Antigen retrieval was done by pretreated tissue sections with 10 mM citrate in a microwave oven for 10 min. Sections were incubated for 2 h with one of the following primary antibodies: rat monoclonal anti-Bax antibody [E63] at a concentration of 1:250 (ab32503; Abcam, Cambridge, U.K.) and TNF-α (dilution 1/100, C07K16/241 Celltech Ltd., U.K.). The sections were rinsed with PBS then incubated with Goat anti-rat IgG H & L (HRP) (ab205718; Abcam, Cambridge, U.K.) for 10 min. The sections were rinsed again with PBS. Finally, sections were incubated 3, 3’-diaminobenzidine tetrahydrochloride (DAB, Sigma, St. Louis Missouri, 63103, U.S.A.). The slides were counterstained with haematoxylin; then mounted. Primary antibodies were replaced by PBS for negative controls.

#### Evaluation of TNF-α and BAX immunostaining

The quantitative immunoreactivity of TNF-α and Bax was evaluated in the liver sections in each group as described by [27], five liver sections were examined. Immuno-reactivity was analyzed in 10 microscopical fields per each section under high-power microscopic field (×400). The percentage of positively stained cells (%) was estimated by color deconvolution ImageJ 1.52 p software (Wayne Rasband, National Institutes of Health, U.S.A.).

## Statistical analysis

The SPSS software program was applied (version 20.0 for windows, IBM). One-way ANOVA setting the probability level to *P*<0.05, post hoc analysis of group differences was performed. Differences between means were calculated by the least significant differences (L.S.D.) test. The treated groups were compared both with each other and with untreated control group. Data were expressed as mean ± SEM.

## Results

### Mortality bioassay and assessment of the insecticidal efficacy of different plant extracts nanoparticles and nanoemulsions

The bioinsecticidal effect of the tested nanoparticles, nanocomposites and nanoemulsions against last instar larvae of *Culex quinquefasciatus* was assessed (Tables 3 and 4) and the efficiency of the same nanomaterials were determined after mosquito pupation (Table 5).

**Table 3:** Concentrations that killed 50% (LC_50_) and 90% (LC_90_) of the tested fourth instar larvae of *Culex quinquefasciatus* treated with four types of nanoparticles, nanocomposites and nanoemulsion in ppm

Used nanomaterials	The LC_50_ and LC_90_ of the tested nanomaterials in ppm		
	LC_50_	LC_90_	Slop (b)
Chitosan	140.98	461.39	0.19
Silver	130.87	389.01	2.57
Chitosn–silver	450.45	860.43	0.42
Lavender	455.67	950.00	0.37

**Table 4:** Coefficient correlation between the applied five concentrations of nanoparticles, nanocomposites and nanoemulsions on pupal mortality^*^

Concentration (ppm)	Chitosan nanoparticles	Silver nanoparticles	Chitosan–silver nanocomopsites	Lavender nanoemulsions
100	1.00^b^	0.60^c^	0.20^d^	0.00^e^
200	1.40^b^	2.40^b^	1.20^c,d^	0.80^d^
400	1.40^b^	3.20^b^	2.00^c^	2.40^c^
800	2.60^ab^	4.40^a^	3.40^b^	3.20^b^
1000	3.80^a^	4.80^a^	4.60^a^	5.00^a^
Control	0.00^c^	0.00^d^	0.00^e^	0.00^e^
L.S.D. _0.05_	1.67	1.15	1.10	0.78

* ANOVA test was significant at 0.05. Means followed by the same letter in each column are not significantly different according to the L.S.D. test.

** Different letters in the same row were significantly different according to the L.S.D. test (p ≤ 0.05).

**Table 5:** Effects of treated fourth instar larvae of *Culex quinquefasciatus* with nanoparticles, nanocomposites, and nanoemulsions of silver, chitosan, chitosan–silver and lavender on percentage of pupation, pupal mortality and adult emergence

Concentrations in ppm	Tested nanoparticles, nanocomposites, nanoemulsions and the measured parameters											
	Chitosan			Silver			Chitosan–silver			Lavender		
	1^*^	2^**^	3^***^	1^*^	2^**^	3^***^	1^*^	2^**^	3^***^	1^*^	2^**^	3^***^
100	60	17	60	88	68	80	84	76	45	90	62	90
200	32	17	20	32	73	30	76	52	100	84	65	100
400	20	100	0.0	8.0	100	0.0	60	17	20	52	70	40
800	12	100	0.0	0.0	100	0.0	32	100	0.0	36	80	0.0
1000	0.0	100	0.0	0.0	100	0.0	8	100	0.0	4.0	96	0.0
Control	100	0.0	100	100	0.0	100	100	0.0	100	100	0.0	100

1^*^= Pupation (%); 2^**^= Pupal mortality (%); 3^***^= Adult emergence (%).

When chitosan nanoparticles were singly tested, 50% (LC_50_) and 90% (LC_90_) of the treated fourth instar larvae have died when exposed to the concentration of 140.98 and 461.39 ppm, respectively (Table 3). Silver nanoparticles were also tested singly against mosquito larvae as an insecticide, reaching LC_50_ and LC_90_ at 130.87 and 389.01 ppm, respectively, with higher lethal effect than chitosan nanoparticles (Table 3). Higher concentrations of the chitosan–silver composite were required to kill 50% and 90% of the treated fourth instar mosquito larvae. These data indicate that either chitosan and/or silver nanoparticles when singly used have more lethal effect on fourth instar mosquito larvae than their composite that kill 50% and 90% of the treated mosquito larvae with higher concentrations of 450.45 and 860.43 ppm, respectively (Table 3).

In contrast, lavender nanoemulsion killed 50% (LC_50_) and 90% (LC_90_) of the treated mosquito larvae at 455.67 and 950.00 ppm, respectively. The present data indicate that lavender nanoemulsion has lower effect than all the tested nanoparticles of chitosan and silver either when used as a single nanoparticle or applied as a nanocomposite (Tables 3 and 4).

As shown in Tables 4 and 5, the highest pupal mortality % was recorded at the three concentrations of 400, 800 and 1000 ppm. The lowest pupal mortality percent (17%) was observed at the lowest concentration of 100 ppm chitosan nanoparticles compared with 0% mortality for the controls (Tables 4 and 5). The present data indicated that the most lethal nanoparticle applied against mosquito larvae and pupae was the silver followed by the chitosan and the chitosan–silver nanocomposites. Meanwhile, the lavender nanoemulsion had the least lethal effect against both mosquito larvae and pupae (Tables 3-5). While the comparison between the LC_50_ and LC_90_ was recorded in Table 3, the coefficient correlation between the four tested nanomaterials was recorded in Table 4.

The pupation (%) of the treated fourth instar larvae decreased as the concentrations of the tested nanomaterials increased. Pupation percent ranged from 60, 84, 88 and 90% when 100 ppm of chitosan, chitosan–silver, silver and lavender nanomaterials were applied respectively. Pupation percent dropped to 8, 4, 0 and 0% when 1000 ppm of chitosan–silver, lavender, chitosan and silver nanomaterials were added, respectively (Table 5).

A remarkable reduction in the mosquito adult emergence was observed when the lowest concentration of 100 ppm of the nanomaterials was applied. The adult emergence was reduced to 45, 60, 80 and 90 when chitosan–silver, chitosan, silver, and lavender nanomaterials were, respectively, applied against the fourth instar larvae of *Culex quinquefasciatus* (Table 5).

### Effect of the tested nanomaterials on the morphology *Culex quinquefasciatus* larvae

**Figure 1:**
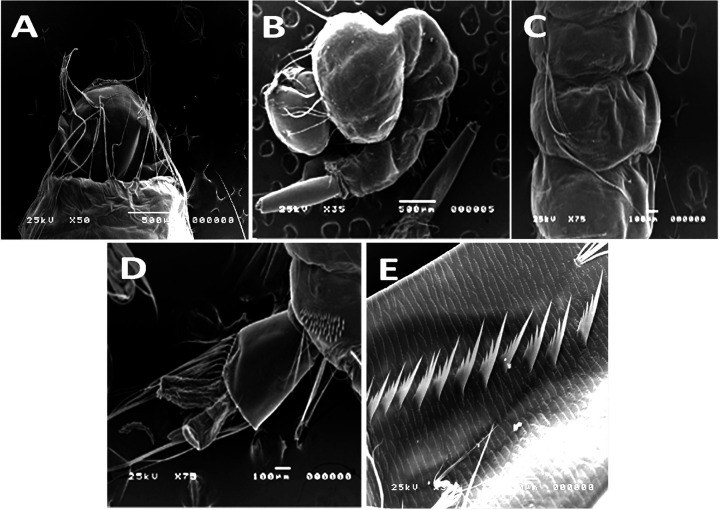
SEM of the normal (control) fourth larval instars of *Culex quinquefasciatus* showing the smooth cuticle surface (**A**) Head and thorax. (**B**) The smooth body surface of the larva. (**C**) Outer body smooth cuticle of abdominal segments. (**D**) The posterior part of the larva showing the anal segment, anal brush, papillae and comb scales. (**E**) Array of pecten-teeth on the siphon.

**Figure 2:**
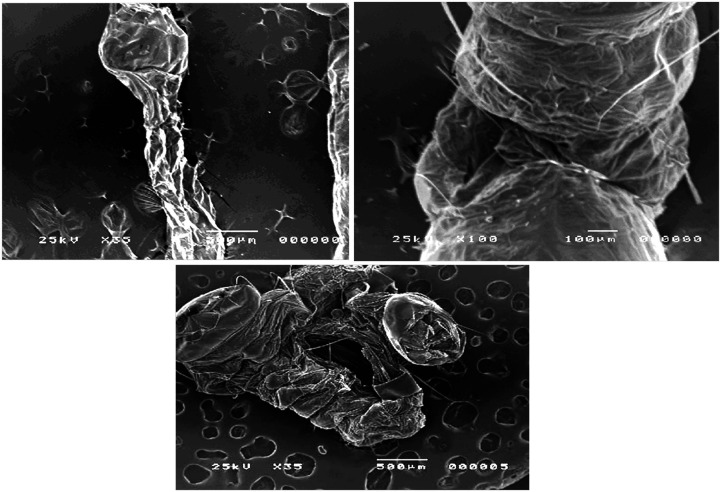
*Culex quinquefasciatus* fourth instar larvae treated with the tested nanomaterials showing different degrees of shrinkage and aberration of the larval cuticle

Different morphological anomalies were recorded on treated larvae by the tested materials. Abnormal changes of treated mosquito larvae including damaged body surface and aberrant cuticle. Different pupal abnormalities including dead, and deformed pupa with different coloration of pupae from dark yellowish to blackish discoloration. The untreated (control) and treated fourth instar larvae of *Culex quinquefasciatus* were examined and photographed by the SEM and showed different malformation. Figure 1 showed photomicrographs of the body surface of the control foruth instar larvae. Meanwhile, the mosquito larvae treated with the nanomaterials had edematous swelling of body parts either in the dorsal and/or the ventral surfaces, their cuticle became erased and/or corrugated while the spines were broken down (Figure 2).

### Evaluation of the toxicity of the tested nanoparticles on rats liver

#### Liver biochemical functions

Data presented in Table 6 indicated that the higher concentrations of the applied nanomaterials caused a significant increase in both ALT and AST enzymes compared with the control rats (*P*<0.05).

**Table 6:** Liver functions of the tested rats post treatment with different concentrations of the nanoparticles of chitosan, silver and their composites, as well as the lavender nanoemulsions in ppm^*^

The tested nanoparticles and nanoemulsions in (ppm)	ALT	AST
Chitosan (500)	39.40^d^	141.75^d^
Chitosan (1000)	46.50^d^	149.70^d^
Silver (500)	63.15^c^	174.05^c^
Silver (1000)	79.40^a^	184.85^a^
Chitosan–silver (500)	58.85^c^	160.35^c^
Chitosan–silver (1000)	70.10^a^	178.75^a^
Lavender (500)	18.90^e^	73.00^e^
Lavender (1000)	24.10^e^	76.30^e^
Control	19.90^e^	83.50^e^
L.S.D._0.05_	10.34	9.81

* ANOVA test was significant at 0.05. Means followed by the same letter in each column are not significantly different according to the L.S.D. test.

** Different letters in the same row were significantly different according to the L.S.D. test (p ≤ 0.05).

The ALT level in the untreated (control) rats was 19.90. When the rats were fed on the higher concentration of chitosan nanoparticles (1000 ppm), the ALT level increased up-to 46.50. While the rats fed on 1000 ppm of silver nanoparticles, the ALT level has reached the highest level of 79.40. When a combination of chitosan and silver nanocomposites was used in a concentration of 1000 ppm, the ALT decreased to the level of 70.10. In comparison, when 1000 ppm of the lavender nanoemulsion was applied, the ALT level has slightly increased from 19.90 in the control to 24.10 (Table 6).

In comparison, the AST level in the untreated (control) rats was 83.50. When rats were fed on the higher concentration of chitosan nanoparticles (1000 ppm), the AST level increased significantly up-to 149.70. Meanwhile, when 1000 ppm of silver nanoparticles were fed to the rats, the AST level reached the highest level of 184.85. When the combination of chitosan–silver nanocomposites was fed to the rats at a concentration of 1000 ppm, the AST increased significantly to the level of 178.75. In comparison, when 1000 ppm of the lavender nanoemulsion was applied, the AST level has slightly decreased from the level of 83.50 in the control to 76.30 (Table 6).

The lavender nanoemulsions were an exception, when used in a concentration of 500 ppm, it significantly decreased both the levels of ALT and AST from 19.90 and 83.50 in the control rats to the level of 18.9 and 73.00 in the treated rats respectively (Table 6). In contrast, when the lavender nanoemulsion concentration of 1000 ppm was applied, only the level of AST was reduced from 83.50 in the control rats to 76.30 in the treated ones (Table 6).

### IL-1β and IL-6 genes expression of treated and untreated rats with different concentrations of nanomaterials

Means of IL-1β in the liver of the rats treated with 500 and 1000 ppm of chitosan nanoparticles were 6.37 and 6.83, respectively. While means of IL-1β in the liver of the rats treated with 500 and 1000 ppm of silver nanoparticles were 8.73 and 12.83, respectively. When 500 and 1000 ppm of nanocomposites of both chitosan and silver were used, the means of IL-1β ranged from 7.67 to 11.23, respectively. When 500 and 1000 ppm of lavender nanoemulsions were applied, the IL-1β levels have slightly increased to the levels of 4.67 and 5.0 above the untreated rats (control) level which was 3.23 (*P*<0.05) (Table 7).

**Table 7:** IL-1β and IL-6 genes expressions in the liver of the rats treated with different concentrations of the tested nanoparticles, nanocomposites and nanoemulsions in ppm^*^

The tested nanomaterials in ppm	IL-1β	IL-6
Chitosan (500)	6.37^e^	4.37^f^
Chitosan (1000)	6.83^d^	6.07^e^
Silver (500)	8.73^c^	8.83^c^
Silver (1000)	12.83^a^	13.63^a^
Chitosan–Silver (500)	7.67^cd^	7.63^cd^
Chitosan–Silver (1000)	11.23^b^	10.33^b^
Lavender (500)	4.67^f^	4.00^f^
Lavender (1000)	5.00^f^	5.17^f^
Control	3.23^g^	3.23^g^
L.S.D._0.05_	1.12	1.38

* ANOVA test was significant at 0.05. Means followed by the same letter in each column are not significantly different according to the L.S.D. test.

** Different letters in the same row were significantly different according to the L.S.D. test (p ≤ 0.05).

In contrast, means of IL-6 in the liver of the rats treated with 500 and 1000 ppm of chitosan nanoparticles were 4.37 and 6.07, respectively. While means of the same gene in the liver of the rats treated with 500 and 1000 ppm of silver nanoparticles were 8.83 and 13.63, respectively. When 500 and 1000 ppm of nanocomposites of both chitosan and silver were applied, the means of IL-6 ranged from 7.63 to 10.33, respectively. When 500 and 1000 ppm of lavender nanoemulsions were applied, the IL-6 levels have slightly increased to the levels of 4.0 and 5.17 above the untreated rats (control) level, which was 3.23 (*P*<0.05) (Table 7).

### Histopathological findings

**Figure 3:**
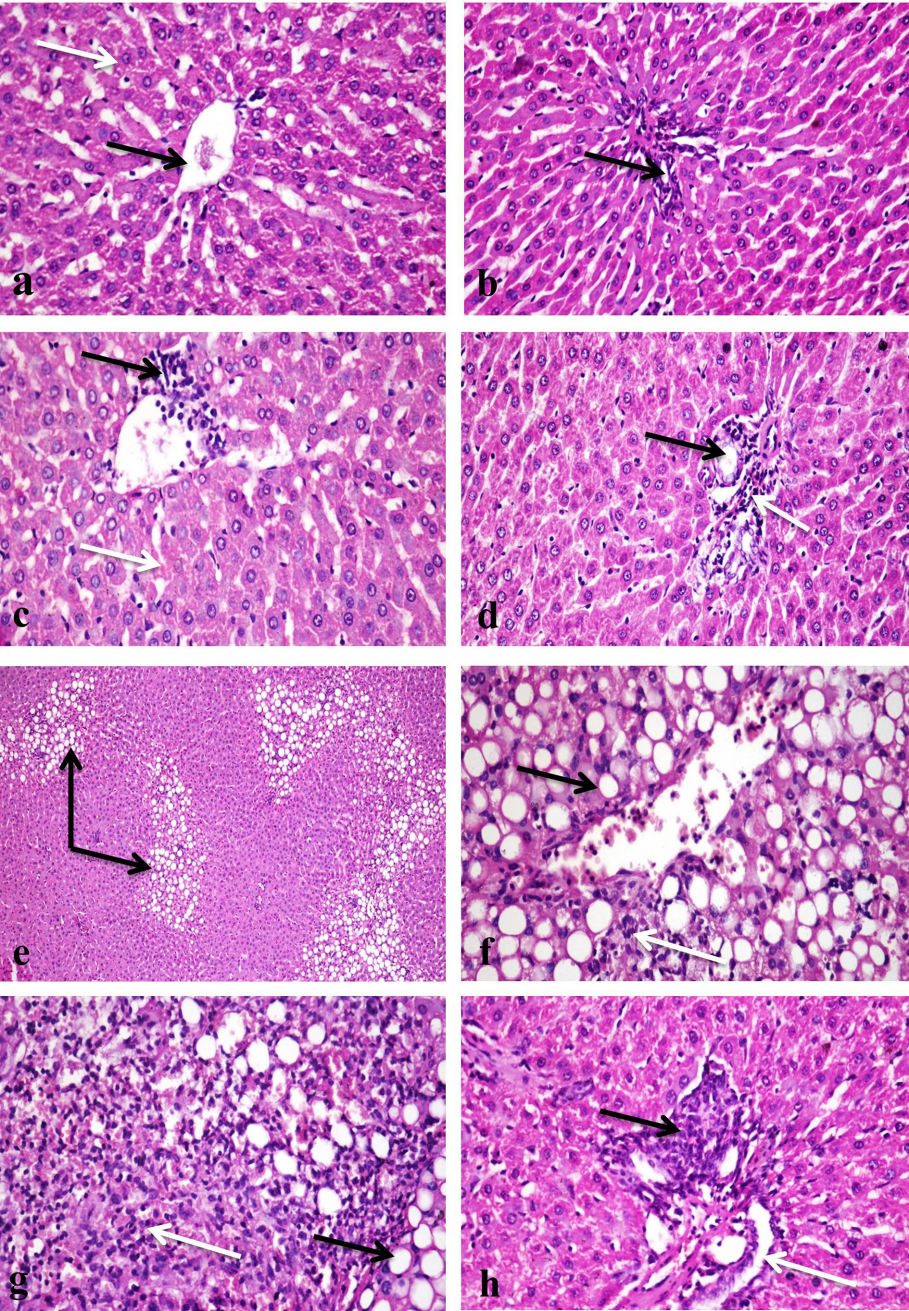
Photomicrograph of a section in the rat liver (**a**) The control group showing normal histological structure of central vein (black arrow) and hepatocytes (white arrow) (H & E ×400). (**b**) The control group showing normal histological structure of portal area (arrow) (H & E ×400). (**c**) A group treated with 500 ppm chitosan nanoparticles showing few mononuclear inflammatory cells infiltration (black arrow) with sinusoidal dilatation (white arrow) (H & E ×400). (**d**) A group treated with 500 ppm chitosan nanoparticles showing hyperplasia of bile duct (black arrow) and mononuclear inflammatory cells infiltration in portal area (white arrow) (H & E ×400). (**e**) A group treated with 1000 ppm chitosan nanoparticles, note multifocal areas of macro- and micro-vesicular steatosis (arrows) (H & E ×100). (**f**) A group treated with 1000 ppm chitosan nanoparticles showing macro-vesicular steatosis (black arrow) with infiltration of mononuclear cells (white arrow) (H & E ×200). (**g**) A group treated with 1000 ppm chitosan nanoparticles showing macro-vesicular steatosis (black arrow), hemorrhage and heavy infiltration of mononuclear cells (white arrow) (H & E ×200). (**h**) A group treated with 1000 ppm chitosan nanoparticles showing mononuclear inflammatory cells infiltration in portal area (black arrow) and ductal hyperplasia (white arrow) (H & E ×400).

Histopathological tests of the liver tissues revealed normal structure in the control untreated rat group (Figure 3a,b). In the rat group treated with 500 ppm chitosan nanoparticle, the liver tissue showed few mononuclear inflammatory cells infiltration with sinusoidal dilatation (Figure 3c). The same group showed hyperplasia of bile duct and mononuclear inflammatory cells infiltration in portal areas (Figure 3d). Multifocal areas of macro- and micro-vesicular steatosis with infiltration of mononuclear cells were detected in the rat group treated with 1000 ppm chitosan nanoparticle (Figure 3e,f), also there were focal areas infiltrated with mononuclear inflammatory cells with presence of hemorrhage (Figure 3g). Proliferation of Kupffer cells with portal areas mononuclear inflammatory cells infiltration with hyperplasia of bile ducts and oval cells proliferation were detected (Figure 3h).

**Figure 4:**
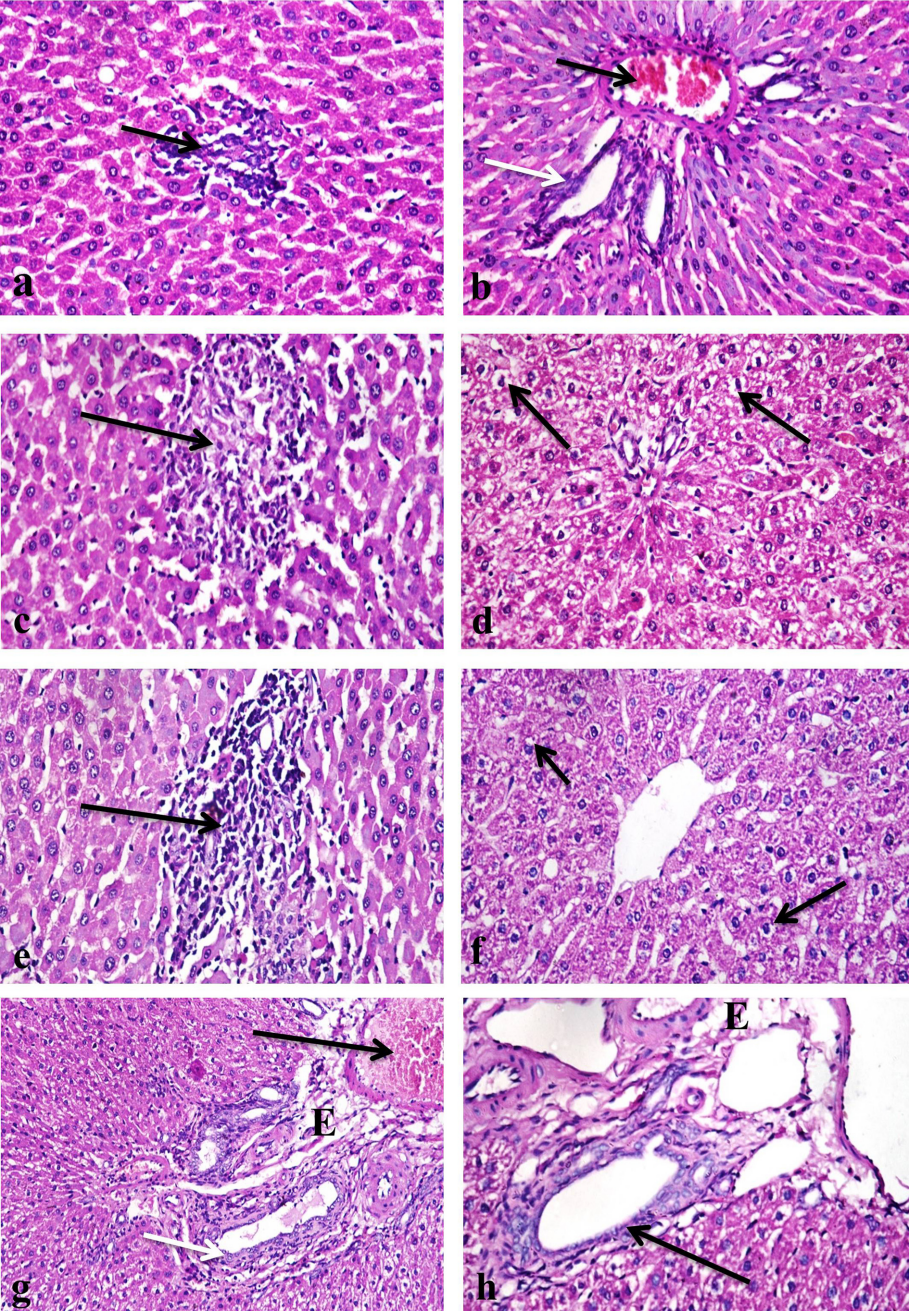
Photomicrograph of a section in the rat liver (**a**) A group treated with 500 ppm silver nanoparticles showing small focal area of mononuclear inflammatory cells infiltration (arrow) (H & E ×400). (**b**) A group treated with 500 ppm silver nanoparticles, note congestion of portal blood vessel (black arrow) with ductal hyperplasia (white arrow) (H & E ×400). (**c**) A group treated with 1000 ppm silver nanoparticles showing focal area of hepatocellular necrosis infiltrated with mononuclear inflammatory cells (arrow) (H & E ×400). (**d**) A group treated with 1000 ppm silver nanoparticles, note vacuolar degeneration of considerable number of hepatocytes (arrows) (H & E ×400). (**e**) A group treated with 1000 ppm silver nanoparticles showing heavy infiltration of portal area with mononuclear inflammatory cells (arrow) (H & E ×400). (f) A group treated with 500 ppm chitosan–silver nanoparticles showing mild vacuolar degeneration of hepatocytes (arrows) (H & E ×400). (**g**) A group treated with 500 ppm chitosan-silver nanoparticles showing congestion of portal blood vessel (black arrow) and ductal hyperplasia (white arrow), note edema of portal area (e) (H & E ×200). (**h**) A group treated with 500 ppm chitosan–silver nanoparticles, note hyperplasia of bile duct (arrow) and edema in portal area (e) (H & E ×400).

The rat groups treated with 500 ppm of nanosilver particles showed small focal areas of hepatocellular necrosis infiltrated with mononuclear inflammatory cells infiltration (Figure 4a). There was also mild sinusoidal dilatation, portal areas showed congestion of portal blood vessels, few inflammatory cells infiltration and ductal hyperplasia with edema dispersing the portal connective tissue (Figure 4b), there was also oval cells hyperplasia. The rats treated with 1000 ppm of nanosilver particles revealed multifocal areas of hepatocellular necrosis infiltrated with mononuclear inflammatory cells (Figure 4c), considerable number of hepatocytes also showed different degrees of vacuolar degeneration with sinusoidal dilatation were also evident (Figure 4d). Kupffer cells proliferation with portal areas infiltration of large number of inflammatory cells, ductal hyperplasia, congestion of portal blood vessels and edema were apparent (Figure 4e). Group treated with 500 ppm chitosan–silver nanocomposites showed mild vacuolar degeneration of hepatocytes (Figure 4f), portal vascular congestion, edema and ductal hyperplasia (Figure 4g,h).

**Figure 5:**
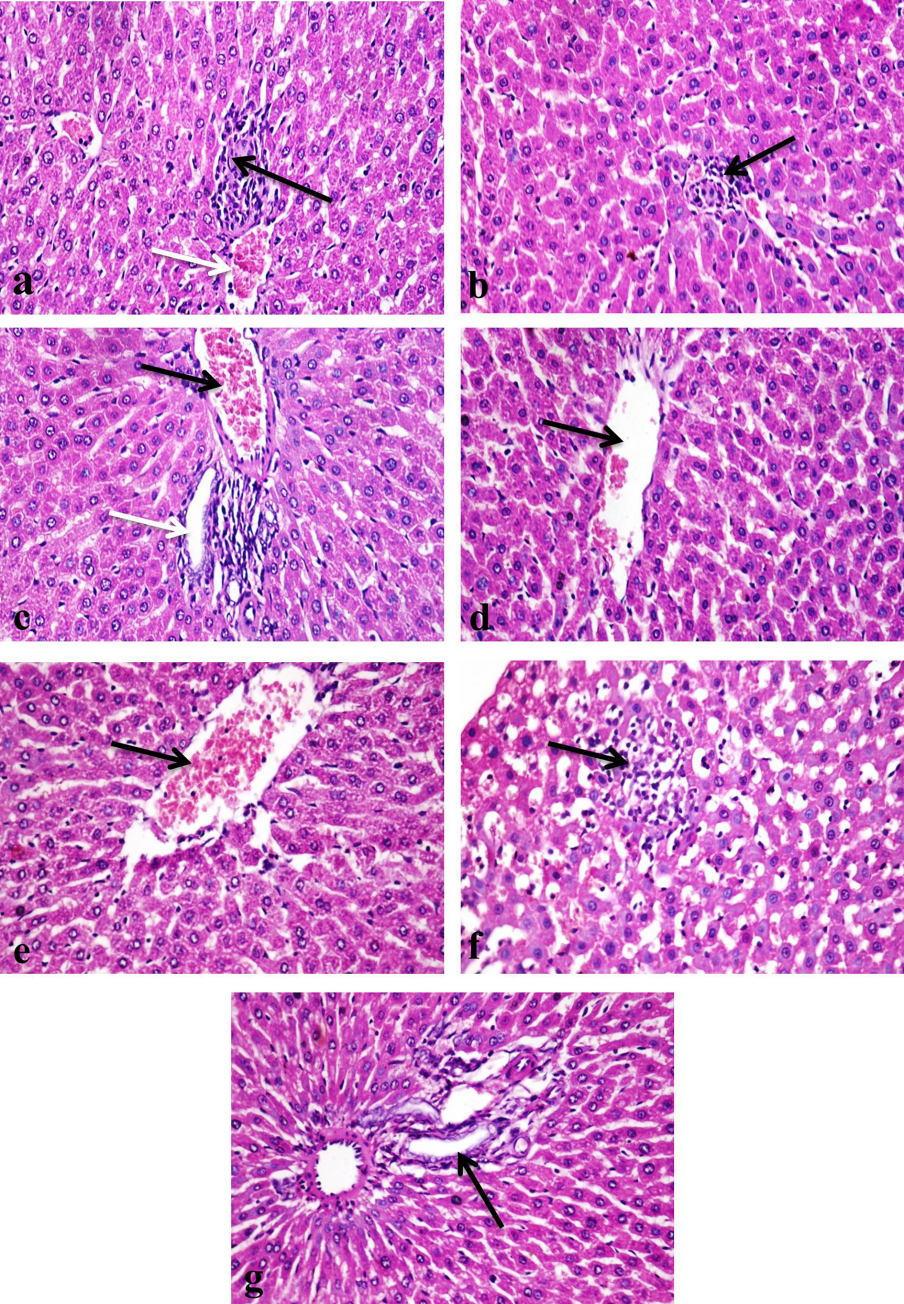
Photomicrograph of a section in the rat liver (**a**) A group treated with 1000 ppm chitosan–silver nanoparticles showing congestion of central vein (white arrow) and focal area of mononuclear inflammatory cells infiltration (black arrow). (**b**) A group treated with 1000 ppm chitosan–silver nanoparticles, note small foci of mononuclear inflammatory cells infiltration (arrow). (**c**) A group treated with 1000 ppm chitosan–silver nanoparticles, note a congestion of portal blood vessel (black arrow) and hyperplasia of bile duct with few mononuclear inflammatory cells infiltration (white arrow). (**d**) A group treated with 500 ppm lavender nanoparticles showing mild congestion of central vein (arrow). (**e**) A group treated with 1000 ppm lavender nanoparticles showing congestion of central vein (arrow). (**f**) A group treated with 1000 ppm lavender nanoparticle, note small focal area of mononuclear infiltration (arrow). (**g**) A group treated with 1000 ppm lavender nanoparticles showing ductal hyperplasia (arrow) (H & E ×400).

In the rat group treated with 1000 ppm of chitosan–silver nanocomposites, hepatocytes revealed mild vacuolar degeneration, congestion of central vein, small focal areas of mononuclear inflammatory cells infiltration (Figure 5a,b), portal areas showed congestion, edema, ductal hyperplasia and few mononuclear inflammatory cells infiltration (Figure 5c). Rat groups treated with 500 and 1000 ppm of lavender nanoemulsion showed mild changes as congestion of central veins (Figure 5d,e) and portal blood vessels, small focal areas of mononuclear inflammatory cells infiltration (Figure 5f) and ductal hyperplasia (Figure 5g).

### Lesion score in the liver of treated rats

All recorded lesions in the treated rat livers were scored according to their severity as shown in Table 8. Several parameters were determined and included: degeneration and necrosis of hepatocytes, mononuclear inflammatory cells infiltration between hepatocytes, Kupffer cells proliferation, sinusoidal dilatation, congestion of central veins, congestion of portal blood vessels, hyperplasia of bile ducts, oval cell hyperplasia, and mononuclear inflammatory cells infiltration in portal areas. Lesions scores were evaluated as follows: score 1 = (<30%), score 2 = (<30–50%), score 3 = (>50%). All control groups had 0.0 score (Table 8). The lesions in tested nanomaterials groups include 500 and 1000 ppm of chitosan and silver nanoparticles, 500 and 1000 ppm of chitosan–silver nanocomposites, and 500 and 1000 ppm of lavender nanoemulsions were scored. Score 3 was detected only when 1000 ppm of chitosan nanoparticles were applied and led to degeneration of hepatocytes and congestion of portal blood vessels. While score 2 was evident when 1000 ppm of chitosan and silver nanoparticles as well as 1000 ppm of lavender nanoemulsions were used and led to necrosis of hepatocytes, Kupffer cells proliferation, Sinusoidal dilatation, and oval cell hyperplasia when 1000 ppm of chitosan nanoparticles were applied. When 1000 ppm of silver nanoparticles were used, the following deterioration was also observed: degeneration and necrosis of hepatocytes, the detection of mononuclear inflammatory cells infiltration between hepatocytes, Kupffer cells proliferation, sinusoidal dilatation, congestion of portal blood vessels, hyperplasia of bile ducts and mononuclear inflammatory cells infiltration in portal areas. Score 2 was also observed when 1000 ppm of lavender nanoemulsion were used and led to congestion of central veins and portal blood vessels. In contrast, score 1 was detected in most of the applied 500 ppm of chitosan and silver nanoparticles as well as the two tested concentrations of chitosan–silver nanocomposites and with the 500 ppm of lavender nanoemulsion. The ‘0’ score was observed with both the lower tested concentrations of nanomaterials and the livers of the control (untreated) rats (Table 8).

**Table 8:** Scoring of histopathological alterations in the liver of all treated rat groups (5 rats each) with the tested nanoparticles and nanoemulsions in (ppm)^*^

Lesions type	Concentrations of the tested nanomaterials in (ppm)							
	Chit.^**^ (500)	Chit. (1000)	Silv.^***^ (500)	Silv. 1000	C-S^@^ (500)	C-S (1000)	Lav.^@@^ (500)	Lav. (1000)
Degeneration of hepatocytes	1.0	3.0	0.0	2.0	1.0	1.0	0.0	0.0
Necrosis of hepatocytes	0.0	2.0	1.0	2.0	0.0	0.0	0.0	0.0
Mononuclear inflammatory cells infiltration between hepatocytes	1.0	3.0	1.0	2.0	0.0	1.0	0.0	1.0
Kupffer cells proliferation	0.0	2.0	0.0	2.0	0.0	0.0	0.0	0.0
Sinusoidal dilatation	1.0	2.0	1.0	2.0	0.0	0.0	0.0	0.0
Congestion of central veins	0.0	1.0	0.0	1.0	0.0	1.0	1.0	2.0
Congestion of portal blood vessels	1.0	3.0	1.0	2.0	1.0	1.0	1.0	2.0
Hyperplasia of bile ducts	1.0	3.0	1.0	2.0	1.0	1.0	1.0	1.0
Oval cell hyperplasia	0.0	2.0	1.0	1.0	0.0	0.0	0.0	0.0
Mononuclear inflammatory cells infiltration in portal areas	1.0	2.0	1.0	2.0	0.0	1.0	0.0	1.0

* The score system was designed as: score 0.0 = absence of any liver lesion in the tested rats, score 1 =(<30%), score 2 =(<30% - 50%), score 3 =(>50%). All control groups had 0.0 score; ^**^Chit. = Chitosan nano-particles;^***^Silv. = Silver nanoparticles; ^@^C-S = Chitosan–silver nanocomposite; ^@@^Lav. = Lavender nanoemulsion.

### Immunohistochemical findings of TNF-α and BAX immunostainng

**Figure 6:**
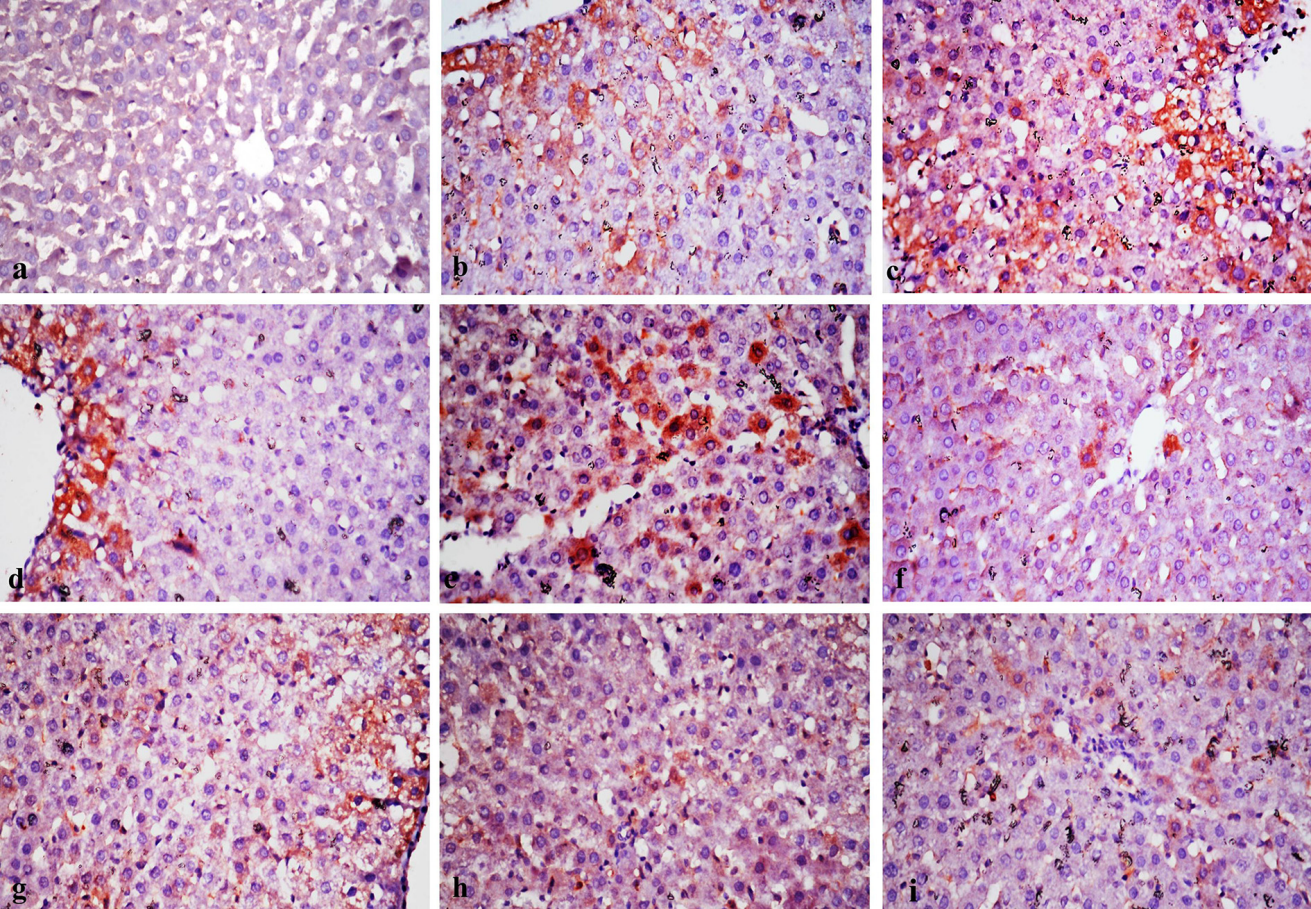
Immunostaining of TNF-α in a section of a rat liver (**a**) The control group showing no TNF-α immune reactive cells in the liver tissue. (**b**) A group treated with 500 ppm chitosan nanoparticles showing weak positive expression of TNF-α. (**c**) A group treated with 1000 ppm chitosan nanoparticles showing strong positive immune expression of TNF-α. (**d**) A group treated with 500 ppm silver nanoparticles showing weak positive expression of TNF-α. (**e**) A group treated with 1000 ppm silver nanoparticles showing strong positive immune expression. (**f**) A group treated with 500 ppm chitosan–silver nanoparticles showing weak expression of TNF-α. (**g**) A group treated with 1000 ppm chitosan–silver nanoparticles showing weak expression of TNF-α. (**h**) A group treated with 500 ppm lavender nanoparticles showing very weak immunoreaction of TNF-α. (**i**) A group treated with 1000 ppm lavender nanoparticles, note very weak immune-expression of TNF-α (TNF-α ×400).

**Figure 7:**
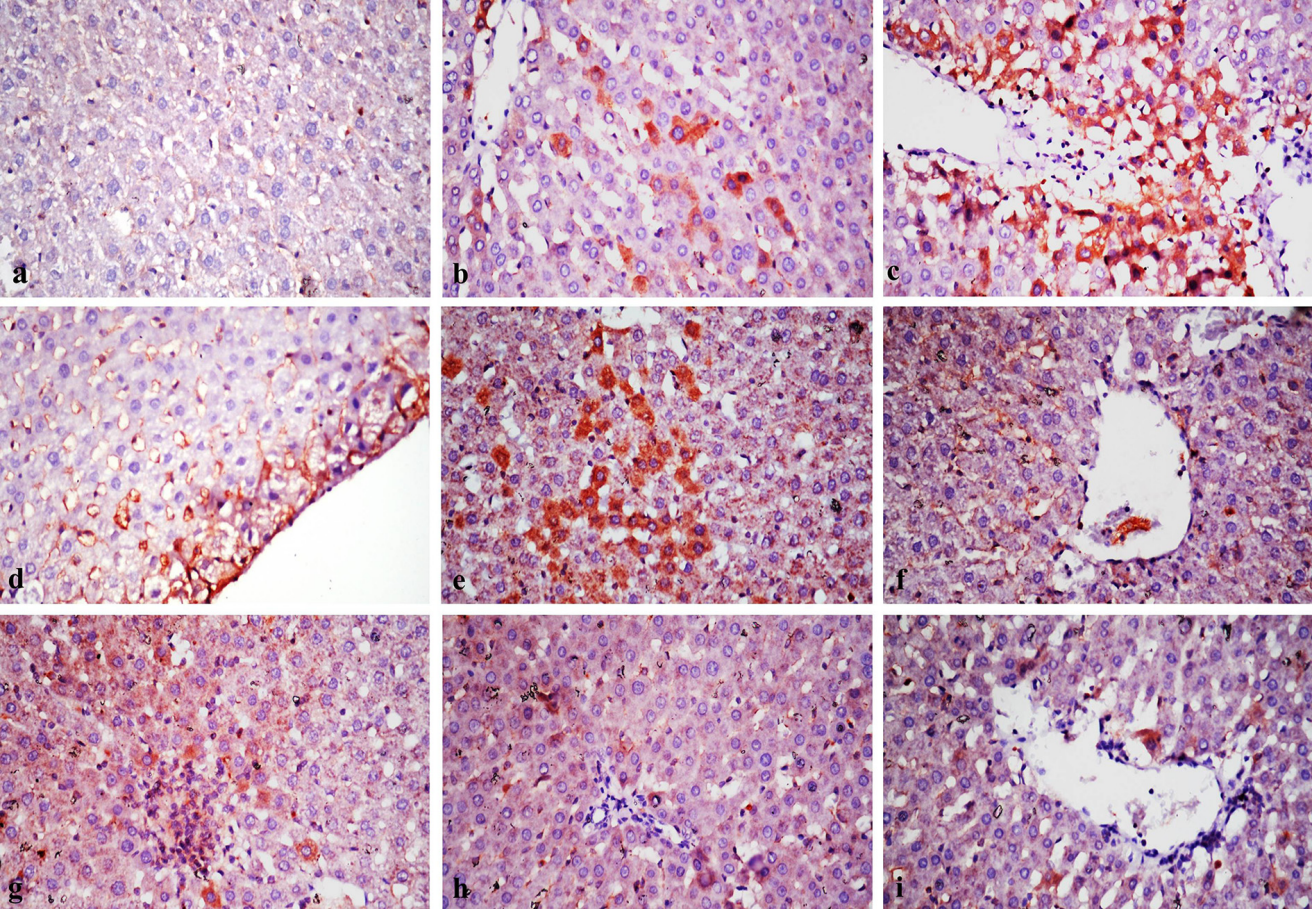
Immunostaining of BAX in a section of a rat liver (**a**) The control rat showing no BAX immune-reactive cells in the liver tissue. (**b**) A group treated with 500 ppm chitosan nanoparticles showing weak positive expression of BAX. (**c**) A group treated with 1000 ppm chitosan nanoparticles showing strong positive immune expression. (**d**) A group treated with 500 ppm silver nanoparticles showing weak positive expression of BAX. (**e**) A group treated with 1000 ppm silver nanoparticles showing strong positive immune expression. (**f**) A group treated with 500 ppm chitosan-silver nanoparticles showing weak expression of BAX. (**g**) A group treated with 1000 ppm chitosan–silver nanoparticles showing weak expression of BAX. (**h**) A group treated with 500 ppm lavender nanoparticles showing very weak immunoreaction of BAX. (**i**) A group treated with 1000 ppm lavender nanoparticles, note very weak immune-expression (BAX ×400)

Immunostaining expression of TNF-α and BAX % area in liver of different treated rat groups was illustrated in Table 9. Immunostaining of TNF-α and BAX in liver revealed no immune-reactive cells in control group (Figures 6a and 7a). Liver tissues in rats treated with both 500 ppm of chitosan and silver nanoparticles revealed weak immune expression of both markers in few hepatocytes (Figures 6b,d and 7b,d). While liver tissues in rats treated with 1000 ppm of chitosan and silver nanoparticles showed strong expression of both BAX and TNF-α in considerable number of hepatocytes (Figures 6c,e and 7c,e). In rat groups treated with either 500 or 1000 ppm of chitosan–silver nanocomposites and lavender nanoemulsions, revealed none or very weak positive immune reactions (Figure 6f,g–i) and (Figure 7f–i).

**Table 9:** Area % of TNF-α and BAX immunostaining expression in liver of experimental rat groups treated with different concentrations of the tested nanomaterials in ppm^*^

	Different tested nanoparticles, nanocomposites and nanoemulsions and their concentrations in ppm^**^							
	Chitosan (500)	Chitosan (1000)	Silver (500)	Silver (1000)	Chitosan–silver (500)	Chitosan–silver (1000)	Lavender (500)	Lavender (1000)
TNF-α	23.2 ± 1.8^b^	48.2 ± 0.8^a^	21.2 ± 2.5^b^	42.3 ± 1.9^a^	13.2 ± 0.4^c^	17.7 ± 0.9^b^	11.3 ± 1.7^c^	11.1.9 ± 2.3^c^
BAX	19.3 ± 2.6^b^	43.1 ± 1.2^a^	18.4 ± 0.9^b^	41.9 ± 2.4^a^	14.3 ± 1.6 ^c^	19.9 ± 1.2^b^	13.7 ± 0.6^c^	12.9 ± 1.2^c^

* Data were expressed as mean ± SE (*n*=5).

** Different letters in the same row were significantly different according to the L.S.D. test (*P*≤0.05).

## Discussion

Nanotechnology opens a new field in several domains including biomedicine, as it acts as antimicrobials agents, catalysts, used in electronics, optical fibers [6].The nanoparticles also used in drug and gene delivery [28], tissue engineering, and parasitic treatment, nanotechnology opens newer pathways for a wide range of applications such as nanomaterials used in eco-friendly products that are cost effective [29,30]. Production of plant-mediated nanoparticles is preferable than chemical and physical approaches because it is less expensive, faster (application with a single step), and does not necessitate high pressure, energy, temperature, and/or the use of highly toxic compounds [31].

Recently, nanoparticles were used as antiparasitic agents against several parasites. Plant-mediated chemicals have been proposed for efficient and quick extracellular synthesis of metal nanoparticles [32], which gave outstanding results as antiplasmodial and mosquitocidal products in the fields [8,29–35]. Recently, the World Health Organization “WHO” [1] has taken action to combat vector-borne diseases by offering evidence-based guidelines for managing vectors and protecting people from infection. Among several produced nanoparticles, silver ones are the most promising and are used in the field of nanomedicines for their antimicrobial activity against different microbial disease agents [36]. The use of silver nanoparticles (AgNPs) as drug carriers is a promising method for the treatment of a wide variety of diseases [37]. Hence, AgNP have emerged with diverse medical applications, including silver based dressings and silver coated medicinal devices, such as nanogels and nanolotions, as well as nanocomposites for mosquitocidal purposes [8–40,29].

At modest dosages, chitosan proved to be harmful to the fourth instar larvae in the laboratory. The LC_50_ values for *An. stephensi*, *Aedes aegypti*, and *Cx. quinquefasciatus* after 24 h of exposure were 114.603, 127.681 and 141.266 g/ml, respectively [15], while in our current results the LC_50_ and LC_90_ were 140.98 and 461.39 ppm, respectively. While the chitosan–silver nanocomposites were highly toxic with LC_50_ and LC_90_ 450.45 and 860.43, less toxic than chitosan-derived AgNPs which were highly toxic, with LC_50_ values of 10.240, 11.349 and 12.426, respectively after 24 h of *Cx. quinquefasciatus* exposure. Similarly, nanosilver structures made from crab shells were extremely poisonous to *An. stephensi*, with LC_50_ values ranging from 3.18 to 6.54 ppm [8].

The primary research work on the use of nanomaterials against mosquitoes concentrated on the larvicidal and pupicidal properties of nanoparticles [37]. Both vectors of malaria and the lymphatic filariasis vector, *Culex quinquefasciatus* was the focus of the study. With few exceptions, green-fabricated metal, metal oxide, silica and carbon nanoparticles have been proven to be more effective than traditional botanical insecticides. The majority of the LC_50_ values calculated on mosquito larvae and pupae varied from 1 to 30 g/ml, according to a recent comprehensive analysis of the toxicity of nanoparticles as larvicidal and pupicidal [2–14].

In the present study, estimation of liver enzymes, ALT and AST levels revealed that higher concentration of chitosan (1000 ppm), create higher ALT levels, while using 1000 ppm silver nanoparticles increases the levels of ALT to reach 79.40. When a combination of both chitosan and silver nanoparticles were used in the same concentration of 1000 ppm, both the ALT and AST slightly decrease to 70.10. Meanwhile, using the lavender nanoemulsion at 1000 ppm has slightly elevated the ALT and lowered the AST below the control level. These results were corroborated by the data recorded in the present histopathological and immunohistochemical studies. The mechanism involved in increasing these enzymes is not clear. Other studies have suggested that such effect may be due to cellular damage or increased plasma membrane permeability [41].

Rats can respond immunologically and upward regulate the expression patterns of different genes in response to external stressors. The liver is the common site of heavy metals toxicity. It is the barrier against different pests, toxic materials and different stressors. To explain the mechanism of innate immunity against external stress factors in rats as a mammalian model as well as to explain the roles of macrophage and lymphocyte which explain different cytokines that secretes several products from mast cells; macrophages and lymphocytes. These products include interferons, interleukins and tumor necrosis factor. Interleukin-1 released in early stage of infection as pro-inflammatory cytokines that regulates both types of immunity (innate and acquired stage); IL-1β was the most potent and fastest genes in humoral immunity that stimulate the inflammation and trigger the immunity. IL-1β originated from liver and kidneys as well as underlying tissues [19–21]. In the present study, we evaluated the expression of the two genes, IL-1β and IL-6, in the rats liver tissues under different concentrations of the tested nanomaterials. The expression of the two genes was greatly affected by the application of chitosan and silver nanoparticles and their nanocomposites, while lavender nanoemulsions have mild effect on the expression of both IL-1β and IL-6 genes when compared with the untreated rats.

In the present study, 500 ppm chitosan nanoparticle treated rat group showed few mononuclear inflammatory cells infiltration with bile duct hyperplasia. Group treated with 1000 ppm chitosan nanoparticle showed macro and microvesicular steatosis of hepatocytes that infiltrated with mononuclear inflammatory cells with hemorrhage, and Kupffer cells proliferation, portal areas showed mononuclear inflammatory cells infiltration with hyperplasia of bile ducts and oval cells hyperplasia. Induced DNA damage in chitosan nanoparticle toxicity is a dose-dependent [42]. Oxidative stress in case of chitosan nanoparticle-induced toxicity is known to participate in tissue injuries, leading to increased production of reactive oxygen species (ROS) as a function of mitochondrial injury only at high concentration [43].

When a group of rats were treated with 500 ppm nanosilver particles, the liver tissues showed small focal areas of hepatocellular necrosis infiltrated with mononuclear inflammatory cells with mild sinusoidal dilatation, portal areas showed congestion, few inflammatory cells infiltration and ductal hyperplasia with edema and oval cells hyperplasia. Higher concentration of 1000 ppm nanosilver treated group revealed multifocal areas of hepatocellular necrosis infiltrated with mononuclear inflammatory cells, there was vacuolar degeneration with sinusoidal dilatation, also there was Kupffer cells proliferation, portal areas revealed infiltration of large number of inflammatory cells, ductal hyperplasia, congestion and edema. Our current results were similar to that described by [44]. These changes observed in liver indicated that the hepatotoxicity of silver nanoparticles is a dose-dependent. Several studies confirmed that the liver is a target organ for the effect of silver nanoparticles [45]. Silver nanoparticles reduce the activity of mitochondria that results in decreased cell energy [44]. In addition, Sardari et al. [46] have cleared the mechanism of silver nanoparticles in liver toxicity, nanoparticles are removed from the liver by macrophages, and the repetition of this process produced higher oxygen radicals. Furthermore, Loghman et al. [47] observed that the toxicity of silver nanoparticles to the mitochondrial activity increased with high used concentrations of silver nanoparticles. They reduce the mitochondrial function, increase membrane leakage, necrosis and induction of apoptosis.

Rat group treated with 500 ppm chitosan-silver nanocomposites showed mild vacuolar degeneration of hepatocytes, portal congestion and edema and ductal hyperplasia, in 1000 ppm chitosan–silver nanoparticles treated group, hepatocytes revealed mild vacuolar degeneration, congestion of central vein, small focal area of mononuclear inflammatory cells infiltration, portal areas showed congestion, edema, ductal hyperplasia and few mononuclear inflammatory cells infiltration. Few researchers affirmed that oral administration of chitosan–silver nanoparticles showed no detectable systemic toxic effect in rats [48]. In other studies, there are no reports regarding the cytotoxicity of CS-AgNPs on cell lines. Cells were exposed to 5–200 μg/ml concentrations of CSAgNPs for 24 h [49]. Cytotoxicity of CS-Ag NPs was a dose-dependent, Oxidative stress is a mechanism by which CS-Ag NPs induce cytotoxicity through enhancing the production of MDA accompanied by depletion of GSH, many physiological and cellular events are induced by oxidative stress, such as inflammation, DNA damage and apoptosis [48].

Rat groups treated with 500 and 1000 ppm of lavender nanoemulsion showed very mild changes in liver, and this result was similar with that previously reported by [50]. The assessment of pathological alteration in the organs of treated animals, both macro and microscopically, is the basis of the safety assessment of *Lavandula angustifolia*, as the oil showed no observable adverse effect [51]. Liver tissues from rats treated with 500 ppm of chitosan and silver nanoparticles revealed weak immune expression of TNF-α and BAX. While liver tissues of rats treated with 1000 ppm chitosan and silver nanoparticles revealed strong expression of both markers. Rat groups treated with 500 and/or 1000 ppm chitosan–silver nanocomposites and lavender nanoemulsions showed nil to very weak positive immune-reactions. TNF-α is a proinflammatory marker and BAX is an apoptotic marker, expression of both markers affirmed that tested nanomaterials produce their adverse effect by oxidative stress and apoptosis by different degrees according to the type of the nanomaterial used and the dose administered. Oxidative stress is a hallmark of inflammation, a mechanism of innate immunity that propagates upon extrinsic or intrinsic stimuli such as toxic nanoparticles. One of the highly important cell type of innate immunity is macrophage, which is known to exert certain functions such as the release of ROS [43].

Conclusion: Green nanoemulsions is more effective than metal or even biodegradable nanoparticles in controlling the mosquitoes *Culex Quinquefasciatus* Say, 1823, this nanoemulsion also proved its safety by toxicological study on rat liver as a non-target mammalian model, as it exerted the least effect on liver function, gene expression, histopathological and immunohistochemical parameters of liver. And this gives future perspectives for use of natural nanoparticles as alternatives for traditional and harmful insecticides, so further studies are suggested to assess the efficacy and safety of natural nanoparticles as insecticides.

## Data Availability

The datasets generated during and/or analyzed during the current study are available from the corresponding author on reasonable request.

## Abbreviations

AgNP: silver nanoparticle
ALT: alanine aminotransferase
AST: aspartate aminotransferase
IL: interleukin
ROS: reactive oxygen species
SEM: scanning electron microscope
